# Promoter of Vegetable Pea *PsPIP2-4* Responds to Abiotic Stresses in Transgenic Tobacco

**DOI:** 10.3390/ijms252413574

**Published:** 2024-12-18

**Authors:** Zhijuan Feng, Na Liu, Yuanpeng Bu, Guwen Zhang, Bin Wang, Yaming Gong

**Affiliations:** Key Laboratory of Vegetable Legumes Germplasm Enhancement and Molecular Breeding in Southern China of Ministry of Agriculture and Rural Affairs, Institute of Vegetables, Zhejiang Academy of Agricultural Sciences, Hangzhou 310021, China; zhijuanke@163.com (Z.F.); ln200811@163.com (N.L.); buyp@zaas.ac.cn (Y.B.); zhangguwen@126.com (G.Z.); wangb@zaas.ac.cn (B.W.)

**Keywords:** vegetable pea, *PsPIP2-4*, promoter, GUS, transgenic tobacco, abiotic stress

## Abstract

Plasma membrane intrinsic proteins (PIPs), one sub-family of aquaporins (AQPs), are responsible for plant abiotic stress responses. However, little information is currently available about the stress responsiveness of the *PIP* promoter in vegetable pea. In the present study, one novel promoter of *PsPIP2-4* which shared high similarity to the *PIP2*-type *AQPs* from other plants, was isolated. Quantitative real-time PCR (qRT-PCR) assays suggested that *PsPIP2-4* was predominantly expressed in leaves and abundantly induced by abiotic stress treatments (polyethylene glycol (PEG) 6000, NaCl, and methyl jasmonate (MeJA)). Further, the promoter activity of *PsPIP2-4* was verified in transgenic tobacco plants. Beta-glucuronidase (GUS) staining driven by the *PsPIP2-4* promoter confirmed that it was mainly detected in the leaves of transgenic seedlings, especially in the guard cells. Exposure of transgenic seedlings to various environmental stimuli proved that the promoter activity of *PsPIP2-4* was abundantly strengthened by osmotic, salt, and MeJA stresses. This research provides one stress-inducible promoter enabling targeted gene expression under abiotic stresses and demonstrates its usefulness in the genetic improvement of plant stress resistance.

## 1. Introduction

Peas (*Pisum sativum* L.), as fresh vegetables and dry grains, are multifunctional crops with great economic value [1,2]. It is rich in carbohydrates, protein, fiber, vitamins, minerals, and phytochemicals, which are beneficial to human health. It serves as the fourth most important legume in the world after soybean, common bean, and chickpea. The yield and quality of peas are frequently compromised due to the environmental stimulus, including osmotic, salt, and cold stresses [3]. It is valuable to discover genes or promoters responsive to environmental stresses and generate stress-resistant varieties for future pea breeding.

AQPs, as trans-membrane transporters of water and small solutes, were key targets in developing stress-resistant crops [4,5]. According to the sequence homology and intracellular location, AQPs were divided into PIPs, tonoplast intrinsic proteins (TIPs), NOD26-like intrinsic proteins (NIPs), small basic intrinsic proteins (SIPs), and the unrecognized X intrinsic proteins (XIPs) [6,7]. Among them, PIPs are generally localized to the plasma membrane, responsible for substrate transport across the cell membrane [8]. In peas, *PsPIP1-1* and *PsPIP2-1* were expressed in the developing seed coat and involved in water transport activity in *Xenopus* oocytes [9]. *PsPIP2-1* also regulated the root and leaf water transports in pea seedlings based on the virus-induced gene silencing (VIGS) method [10]. Recently, nine *PsPIPs* were identified that were transcriptionally regulated by the nanoantioxidant fullerol in imbibing embryos under osmotic stress [11]. However, the experimental evidence on their precise roles in response to abiotic stresses is still scarce.

Promoter, as an important molecular switch, was essential to drive the transcription of gene expression [12,13]. *PIP* promoters were involved in controlling multiple biological processes. In *Arabidopsis*, the promoter of *AtPIP2;7* functioned in the leaves, roots, and cotyledons and responded to salt stress [14]. The promoters of *AtPIP1;4*, *AtPIP2;5*, and *AtPIP2;6* directed the expression profiles specific to the base of leaf petioles and part of the flowers [15]. In rice, the promoter of *OsPIP1;3* was highly expressed in the radicles of germinated seeds and embryos [16]. In *Brassica napus*, the promoter activity of *BnPIP1* was distributed primarily to the vascular systems and tissues with rapidly expanding and proliferating cells [17]. In rose, the promoter activity of *RhPIP2;1* was inhibited by NaCl, cold, 1-aminocyclopropane-1-carboxylate (ACC), and ABA in the roots and increased by mannitol and gibberellin A3 (GA3) in the rosettes [18]. In cotton, the promoter of *GhPIP2;7* was active in the leaves and responsive to mannitol stress [19]. In banana, the activity of the *MaPIP1;1* promoter was intense in the roots and faint in the leaves after mannitol-induced osmotic stress [20]. Nevertheless, the regulatory roles of *PIP* promoters under abiotic stresses in pea remain unclear.

In this research, one novel promoter of *PsPIP2-4* which belonged to the *PIP2*-type *AQP*, was isolated from the vegetable pea genome. Expressional patterns of *PsPIP2-4* in different tissues (leaves, roots, and stems) and in response to various abiotic stresses PEG6000, NaCl, and MeJA were evaluated by qRT-PCR. The promoter elements of *PsPIP2-4* were analyzed, and its promoter activity was validated using the *GUS* reporter gene in transgenic tobacco plants under stress conditions. The results will provide a theoretical basis for the utilization of the *PsPIP2-4* promoter and exploration of the regulation mechanism of *PsPIP2-4* in response to abiotic stresses.

## 2. Results

### 2.1. Structural Analysis of PsPIP2-4

**Figure 1:**
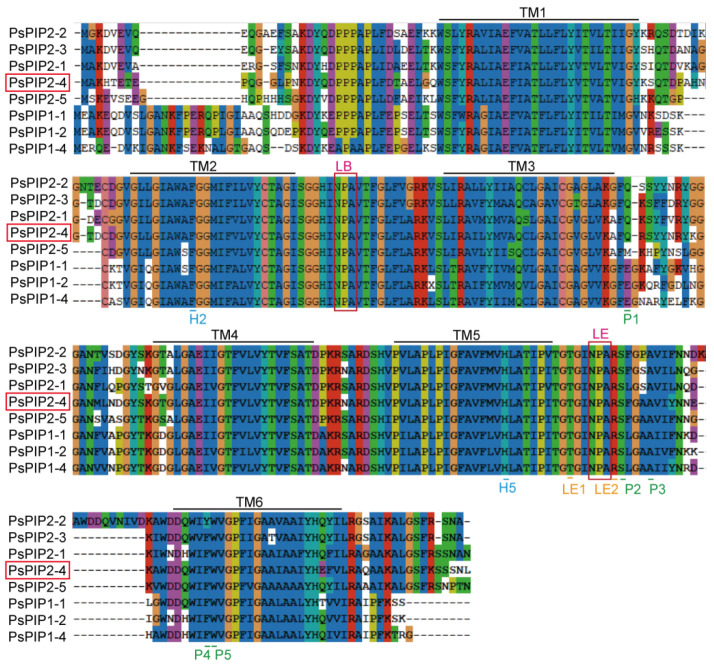
Conserved TM domains and amino acid residues (NPA motifs, ar/R filters, and FPs) of PsPIP2-4. TM1, TM2, TM3, TM4, TM5, and TM6 represent the TM domains. LB and LE represent the NPA motifs. H2, H5, LE1, and LE2 represent the ar/R filters. P1, P2, P3, P4, and P5 represent the FPs. Amino acid residues with the some color represent the high conserved amino acids. PsPIP2-4 is marked with the red box.

To identify the structural characteristics of PsPIP2-4, multiple alignments of PsPIPs were conducted. It was found that PsPIP2-4 contained six trans-membrane (TM) domains (TM1, TM2, TM3, TM4, TM5, and TM6), two Asn-Pro-Ala (NPA) motifs (LB and LE), aromatic/arginine (ar/R) selectivity filters (H2, H5, LE1, and LE2) of F-H-T-R, and Froger’s positions (FPs) (P1, P2, P3, P4, and P5) of Q-S-A-F-W (Figure 1).

### 2.2. Phylogenetic Analysis of PsPIP2-4

**Figure 2:**
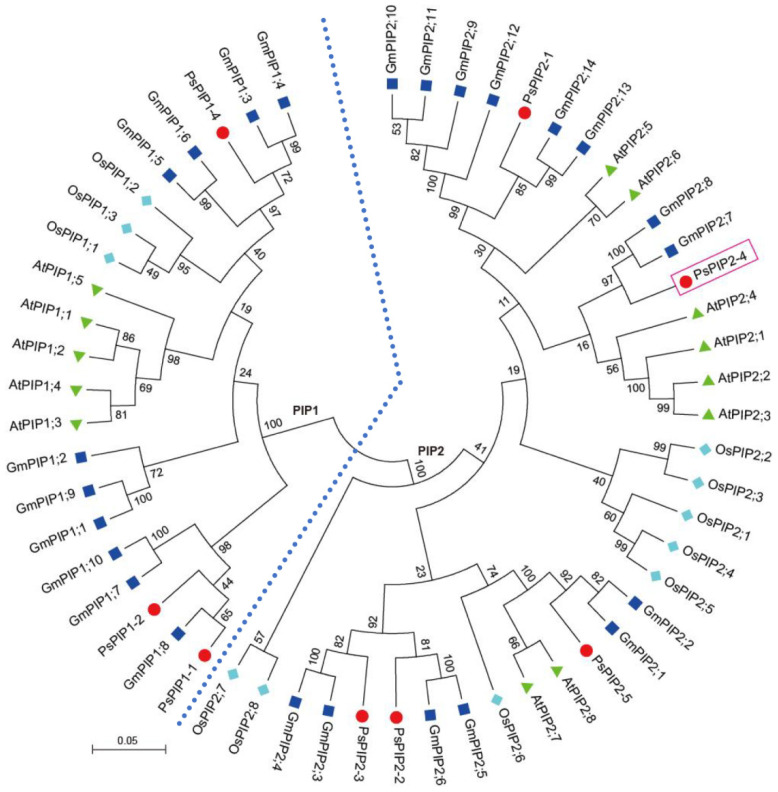
Phylogenetic classification of PIP proteins from *Pisum sativum*, *Glycine max*, *Arabidopsis thaliana*, and *Oryza sativa*. PsPIPs, GmPIPs, AtPIPs, and OsPIPs are marked with the red circle, dark blue square, green triangle, and light blue diamond, respectively. The tree is classified into two groups of PIP1 and PIP2, which are differentiated with the light blue dashed line. PsPIP2-4 is marked with the red box.

To clarify the evolutionary relationship of PsPIP2-4, a phylogenetic tree was generated using *Pisum sativum* PsPIPs, *Glycine max* GmPIPs, *Arabidopsis thaliana* AtPIPs, and *Oryza sativa* OsPIPs. It was indicated that PsPIPs were divided into two sub-families: PIP1 and PIP2. PsPIP2-4, belonging to the PIP2 sub-family, was highly homologous to soybean GmPIP2;7 and GmPIP2;8 and *Arabidopsis* AtPIP2;1, AtPIP2;2, AtPIP2;3, and AtPIP2;4 (Figure 2).

### 2.3. Expression Pattern Analyses of PsPIP2-4 in Different Tissues and in Response to Different Abiotic Stresses

**Figure 3:**
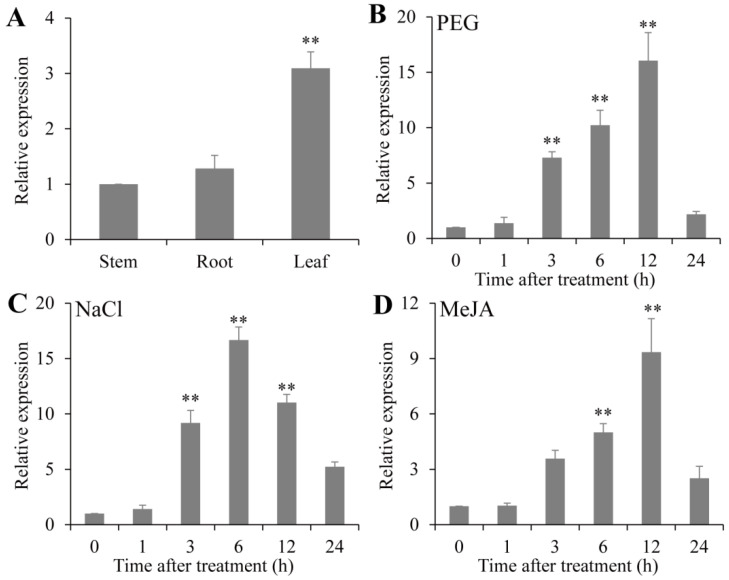
Expression profiles of *PsPIP2-4* under 15% PEG, 200 mM NaCl, and 100 µM MeJA treatments in vegetable pea seedlings. Expression patterns of *PsPIP2-4* in leaves, roots, and stems (**A**). Expression profiles of *PsPIP2-4* under 15% PEG (**B**), 200 mM NaCl (**C**), and 100 µM MeJA (**D**) stresses in the whole vegetable pea plants. Furthermore, 0, 1, 3, 6, 12, and 24 h represent the treatment times. ** represents means of six replicates (*n* = 6) that are significantly different at the *p* < 0.01 level (*t*-test).

To explore the expression profiles of *PsPIP2-4*, qRT-PCR assays for *PsPIP2-4* in different tissues and under different stress treatments in vegetable pea seedlings were conducted. In different tissues, *PsPIP2-4* was more significantly expressed in leaves (3.1-fold) than in roots and stems (Figure 3A). After 15% PEG treatment, *PsPIP2-4* was strongly up-regulated at 3 h, 6 h, and 12 h (7.3-fold, 10.2-fold, and 16.0-fold) (Figure 3B). When exposed to 200 mM NaCl treatment, the expression of *PsPIP2-4* showed a dramatic increase at 3 h, 6 h, and 12 h (9.1-fold, 16.6-fold, and 11.0-fold) (Figure 3C). For 100 µM MeJA treatment, the expression of *PsPIP2-4* was sharply induced at 6 h and 12 h (5.0-fold and 9.3-fold) (Figure 3D).

### 2.4. Isolation of the PsPIP2-4 Promoter

**Figure 4:**
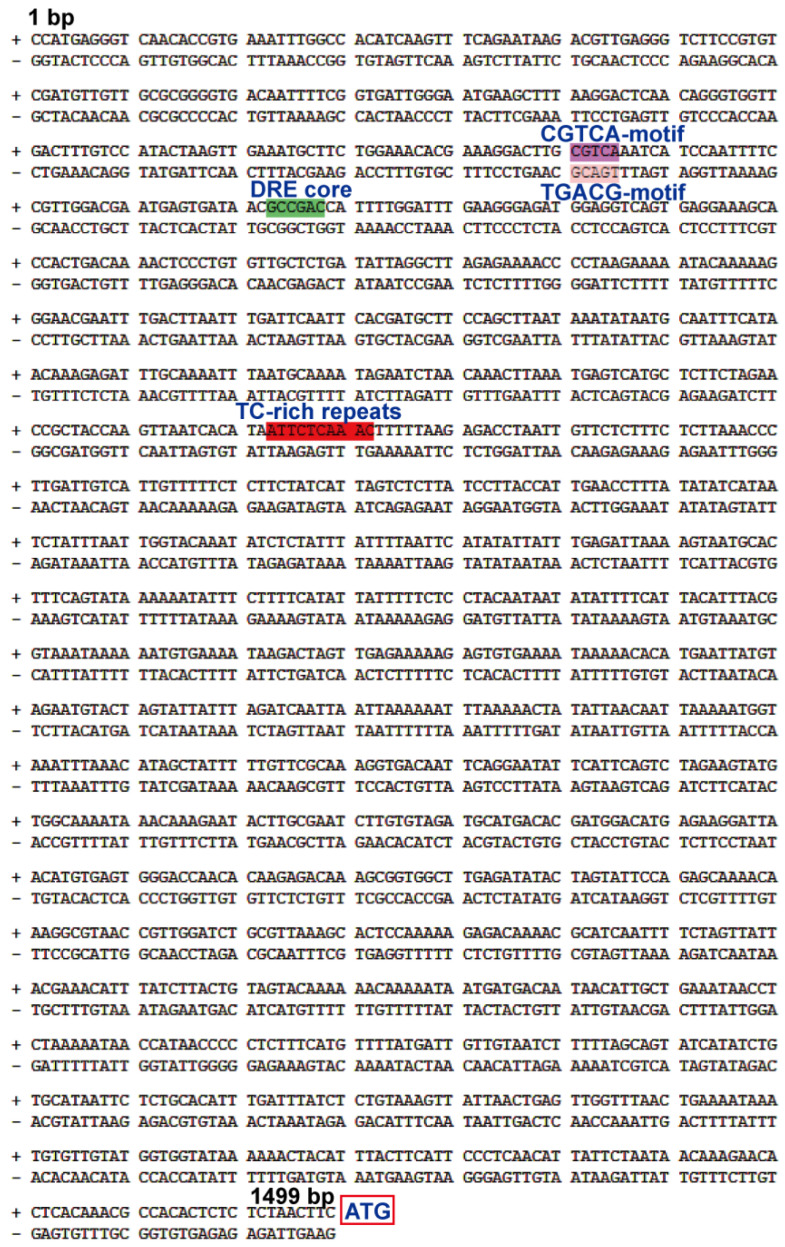
*Cis*-acting element composition of the *PsPIP2-4* promoter. The different colored shades represent different elements. + and − represent the positive and negative strands of the *PsPIP2-4* promoter, respectively. Furthermore, 1 and 1499 bp represent the orientation of the *PsPIP2-4* promoter. The red box represents the ATG start codon of the *PsPIP2-4* gene.

Furthermore, the promoter sequence of *PsPIP2-4* was amplified based on the annotated vegetable pea genome. Its length was 1.499 kb, upstream of the ATG start codon of *PsPIP2-4*. Using plantCARE, one drought stress regulatory element (DRE core), one defense and stress regulatory element (TC-rich repeats), and one MeJA regulatory element (CGTCA or TGACG motif) were identified in the *PsPIP2-4* promoter (Table 1; Figure 4).

### 2.5. GUS Activities of the PsPIP2-4 Promoter in Response to Osmotic, Salt, and MeJA Stresses

**Figure 5:**
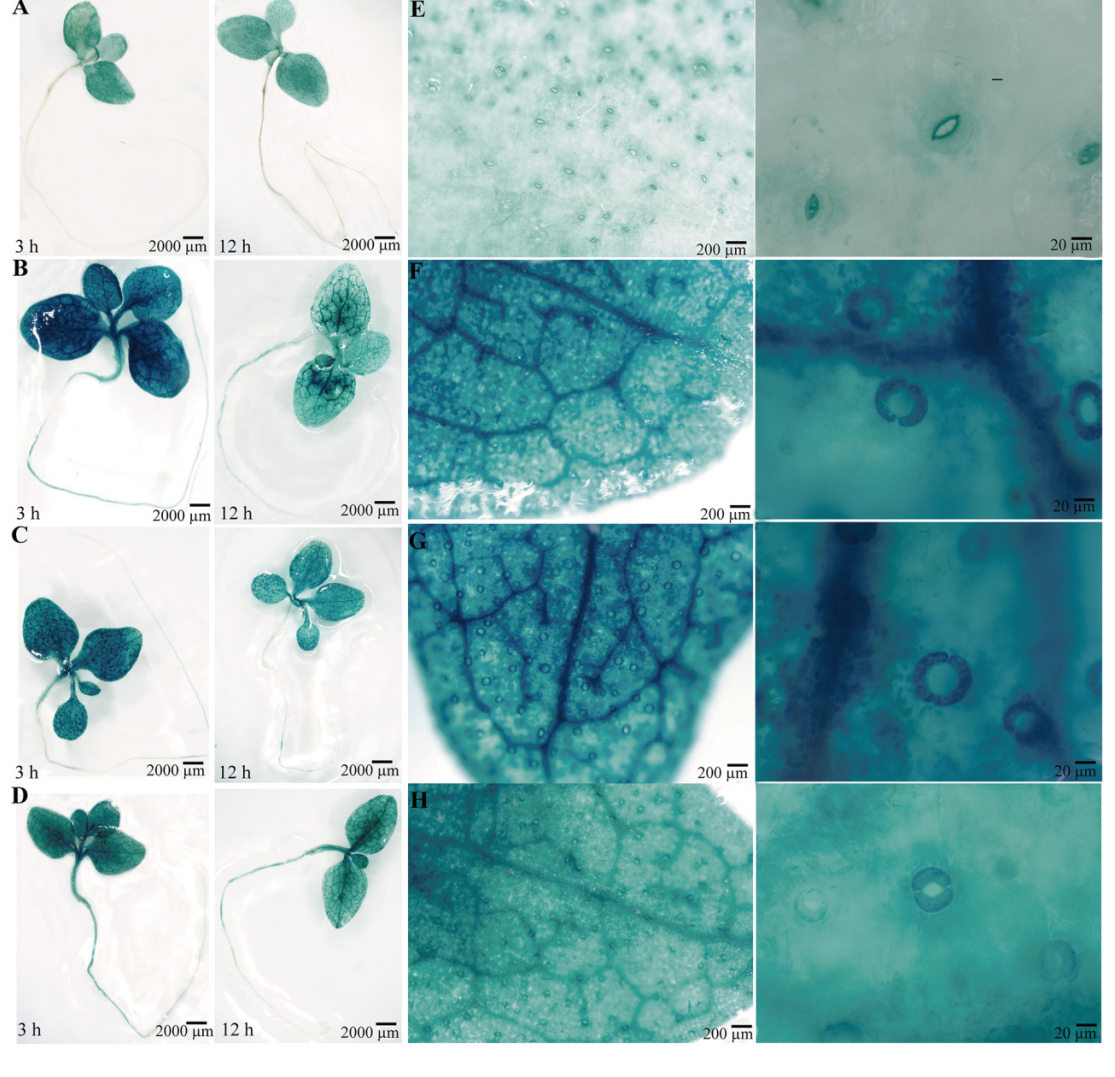
GUS histochemical staining for the *PsPIP2-4* promoter after 6% PEG6000, 100 mM NaCl, and 100 µM MeJA stress treatments in transgenic tobacco seedlings. GUS staining for the whole transgenic plants under normal (**A**), 6% PEG (**B**), 100 mM NaCl (**C**), and 100 µM MeJA (**D**) stress conditions for 3 h and 12 h. GUS staining for the transgenic leaves and their guard cells under normal (**E**), 6% PEG (**F**), 100 mM NaCl (**G**), and 100 µM MeJA (**H**) stress conditions for 3 h.

**Figure 6:**
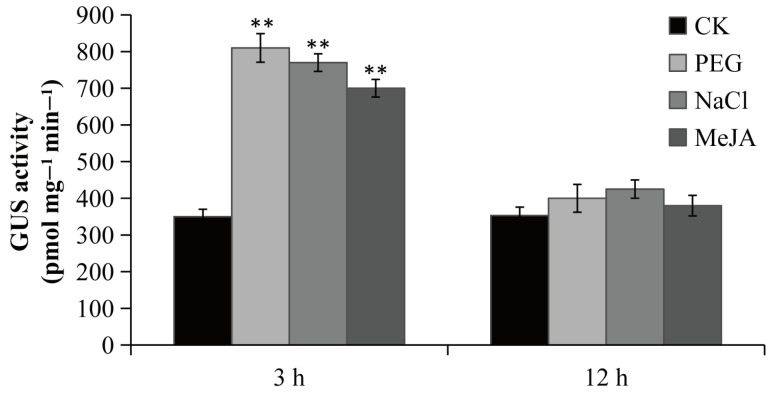
GUS activities of the *PsPIP2-4* promoter after 6% PEG6000, 100 mM NaCl, and 100 µM MeJA stress treatments in the whole transgenic tobacco seedlings. GUS activities of seedlings without stress treatments at the same time points under the same environmental conditions are used as a control (CK). ** represents means of nine replicates (*n* = 9) that are significantly different at the *p* < 0.01 level (*t*-test).

To confirm the function of the *PsPIP2-4* promoter, it was fused to the *GUS* reporter gene and introduced into tobacco. Positive transgenic tobacco that contained the targeted promoter region of *PsPIP2-4* were subjected to GUS staining and activity analyses (Figure S1). Under normal growth conditions, the negative (WT) and positive control (pCAMBIA1300-pBI101) tobacco seedlings could not be stained (Figure S2A,B). In the parallel experiment, GUS staining driven by the *PsPIP2-4* promoter was present in the leaves of transgenic seedlings (Figure 5A). After 6% PEG6000 or 100 mM NaCl treatment for 3 h, GUS staining of the *PsPIP2-4* promoter was highly increased in the leaves, stems, and roots of transgenic seedlings (Figure 5B,C). The similar change of GUS staining was exhibited in the 100 µM MeJA-treated transgenic seedlings (Figure 5D). In addition, strong GUS staining was displayed in the guard cells of leaves of transgenic seedlings (Figure 5E–H). GUS protein detection analyses also verified that the activities of the *PsPIP2-4* promoter were remarkably elevated after osmotic, salt, and MeJA treatments for 3 h (Figure 6).

## 3. Discussion

Pea played a critical role in the discovery of Mendel’s laws of inheritance in the 18th century [21]. Recently, the genome research for pea had made significant breakthroughs, and many functional genes were identified from the annotated genome [22,23,24]. With high nutritional value, the demand of consumers for peas continued to rise [25]. By contrast, rapidly changing climate severely affected the yield and quality of peas. It was urgent to mine genetic resources for improving the stress resistance of peas.

PIPs, known as plasma membrane AQPs, were crucial for plants to combat multiple environmental stimuli [26]. Various species have different PIP members with diverse structural characteristics. Protein structure analyses showed that PsPIPs contained conservative TM domains, NPA motifs, and Ar/R selectivity filters, and divergent FPs (Figure 1). Distinct FPs determined the transport specificities, which contributed to the neo-functionalization and complex regulation modes for PIPs [27,28]. Evolutionary analyses indicated that PsPIPs could be classified into two groups: PsPIP1s and PsPIP2s (Figure 2). The main structural differences between PsPIP1s and PsPIP2s were the length and amino acid composition of the N and C termini, as shown in Figure 1. It was reported that PIP2s generally mediated significant trans-membrane water transport, while most PIP1s could not [29,30,31,32,33]. Additionally, PsPIPs exhibited a closer phylogenetic relationship with GmPIPs and AtPIPs than OsPIPs. In soybeans, it had been proved that GmPIP1;6 and GmPIP2;9 conferred drought and salt resistance [34,35]. In *Arabidopsis*, AtPIP2;1 regulated the seed germination following salt stress [36]. In the present study, PsPIP2-4 showed the highest similarity to the known PIP2-type protein of AtPIP2;1 (Figure 2), which might function in response to abiotic stresses. Mining the key PsPIP members controlling abiotic stress tolerance became increasingly significant.

Gene expression patterns could be used to investigate the biological functions of specific genes [37,38]. Many reviews pointed out that *PIPs* represented the large amplitude of variation in expression [4,5,6,7]. For diverse tissues, some *PIPs* were expressed in various tissues, and individual *PIPs* were expressed in specific tissues. For abiotic factors, some transcripts of *PIPs* were highly expressed while others were weakly expressed. Different spatio-temporal expression patterns devoted to discerning the diversification and redundancy among *PIP* members [39]. For *PsPIP2-4*, it possessed relatively high expression in leaves and remarkable expression changes in response to osmotic, salt, and MeJA stresses (Figure 3), implying its potential application in the cultivation of stress-resistant pea.

Gene expression was quantitatively modulated by the specific promoter, which contained various *cis*-acting elements [40]. In many species, multiple abiotic stress-related elements in promoters of *PIPs* have been discovered. The *PsPIP2-4* promoter harbored the regulatory elements of TC-rich repeats, DRE core, CGTCA, and TGACG motifs (Figure 4), which were responsible for moderating its gene expression. Previously, these regulatory elements have been investigated in multiple plant species [41,42,43]. In transgenic tobacco seedlings, the *PsPIP2-4* promoter conferred intense GUS expression in the leaves, especially in the guard cells (Figure 5), which regulated the opening and closure of stomata. A similar result was observed in *Arabidopsis AtPIP2;1* and maize *ZmPIP2;5,* which regulated the stomata movements [44,45]. Stomatal movements resulted from turgor changes in the guard cells, and PIPs in the plasma membrane were required for these rapid cell volume fluctuations [46,47,48,49]. Quantitative analyses of GUS activities verified the *PsPIP2-4* promoter was strengthened by osmotic, salt, and MeJA stresses (Figure 6), which was in line with its expression patterns. Accordingly, the *PsPIP2-4* promoter was one stress-inducible promoter, mediating osmotic, salt, and MeJA stresses. Inducible or tissue-specific promoters could restrict the gene expression under certain conditions or in specific tissues, thus eliminating the abnormal phenotypes with the use of constitutive promoters. Current data suggested the *PsPIP2-4* promoter could be exploited as a novel promoter to accurately direct targeted transgene expression across both time and space in plant stress resistance genetic engineering.

## 4. Materials and Methods

### 4.1. Vegetable Pea Stress Treatment

Zhewan 3, which was one of the Zhewan series vegetable pea cultivated varieties and resistant to osmotic stress, was used in this study. Among the Zhewan series of vegetable pea cultivated varieties, the high-quality reference genome of Zhewan 1 has been assembled and published, due to the high yield and quality [24]. The uniformly germinated seeds of the vegetable pea cultivated variety Zhewan 3 were planted in the round pot (diameter, 11 cm) containing 135 g of soil mixture (nutrient soil and vermiculite in a 3:1 ratio) within the controlled artificial climate chamber (16 h light cycle at 22 °C; and 8 h dark cycle at 20 °C; relative humidity of 60%; and light intensity of 25,000 Lux). To ensure uniform growth conditions, each pot was administered 30 mL of water daily, and the soil moisture was maintained at levels within 80–85% of the maximum soil water holding capacity by the gravimetric method with daily weighing experiment as previously reported [50,51]. For stress treatments, the root systems of 25-day-old vegetable pea seedlings with the sixth compound leaf appearing and the sixth stipule fully spreading out were removed from the soil and soaked in the glass containers containing 200 mL solutions with 15% PEG6000, 200 mM NaCl, and 100 µM MeJA. The stress-treated vegetable pea seedlings were cultivated under the same environmental conditions, and whole seedlings were collected at 3 h, 6 h, 12 h, or 24 h after stress treatments. In the parallel experiment, non-stressed vegetable pea seedlings that were steeped in the glass containers containing 200 mL of water at the same time points under the same environmental conditions were used as controls. Under these stress concentrations, obvious differences in phenotype between the control and stress-treated vegetable pea seedlings in wilting were observed. Leaves, roots, and stems from non-stressed vegetable pea seedlings were obtained for gene expression analysis in different tissues. All samples of vegetable pea in the treatment and control groups were rapidly dropped into liquid nitrogen to maintain sample integrity before storage at −80 °C until RNA and DNA extraction assays.

### 4.2. Phylogenetic Analysis

PIP protein sequences of *Pisum sativum*, *Glycine max*, *Arabidopsis thaliana*, and *Oryza sativa* were obtained as reported previously [52,53,54]. Multiple sequence alignments were performed with ClustalX2. A phylogenetic tree was generated based on the alignment results by the bootstrap neighbor-joining (NJ) method in MEGA7.0 (bootstrap number set to 1000).

### 4.3. QRT-PCR

RNA extraction of vegetable pea was accomplished according to the RNAprep Pure Plant Kit (Tiangen, Beijing, China). Reverse transcription PCR was conducted using the TransScript First-Strand cDNA Synthesis SuperMix (Transgen, Beijing, China). The synthesis of cDNA was carried out with reference to the FastQuant RT Kit (Tiangen, Beijing, China). Specific qRT-PCR primers with the forward primer (5′-AATCACAAACCGATCCAGC-3′) and the reverse primer (5′-CCTAAACATTGAGCCACCATG-3′) were designed by Primer 5.0 for *PsPIP2-4* as listed in Table S1. For normalization, two reference genes of *Psβtubulin* and *PsEF1α* were utilized as the internal controls, respectively [24,55,56]. The qRT-PCR reaction was conducted with the SuperReal PreMix Plus SuperReal (SYBR Green) (Tiangen, Beijing, China) on the Applied Biosystems StepOnePlusTM Real-Time System according to the protocol provided by the manufacturer. The qRT-PCR programs for *Psβtubulin*, *PsEF1α*, and *PsPIP2-4* were set as follows: initial denaturation at 94 °C for 30 s and 40 cycles of denaturation at 94 °C for 10 s, primer annealing at 55–58 °C for 30 s, and extension at 72 °C for 30 s. Each experiment was performed with three biological duplications. The technique of 2^−ΔΔCT^ was used to assess the gene relative transcript levels [57,58,59]. The means of six replicates for gene relative transcript levels of *PsPIP2-4* in different tissues and in response to different abiotic stresses were calculated by Student’s *t*-test at a significant level of *p* < 0.01.

### 4.4. Promoter Isolation

Genomic DNA of vegetable pea was extracted with the CTAB method. The promoter sequence of *PsPIP2-4* was isolated from the vegetable pea genome DNA with the PrimeSTAR^®^ GXL DNA Polymerase (TaKaRa, Dalian, China) by PCR according to the manufacturer’s instructions. Specific PCR primers with the forward primer (5′-CCATGAGGGTCAACACCGTG-3′) and the reverse primer (5′-GAAGTTAGAGAGAGTGTGG-3′) designed by the Primer 5.0 for the *PsPIP2-4* promoter were provided in Table S1. The PCR amplification product was attached to the cloning vector pCE2 TA/Blunt (Vazyme, Nanjing, China) as directed by the manufacturer and then sequenced for validation. The correct promoter sequence of *PsPIP2-4* was blasted against the recently published vegetable pea reference genome of the Zhewan series’ main cultivated variety Zhewan 1 [24]. The detailed sequence information of the *PsPIP2-4* promoter was present in Dataset S1. *Cis*-acting elements present in the *PsPIP2-4* promoter were analyzed by the online tool of PlantCARE (http://bioinformatics.psb.ugent.be/webtools/plantcare/html/, accessed on 20 June 2022) with default settings [60].

### 4.5. Transgenic Tobacco Generation

To create the proPsPIP2-4::GUS fusion construct, the promoter of *PsPIP2-4* was inserted into *Pst* I/*BamH* I restriction enzyme cleavage sites upstream of the *GUS* reporter gene of the pCAMBIA1300-pBI101 vector (Figure S3) using the restriction endonucleases *Pst* I/*BamH* I and T4 DNA ligase (TaKaRa, Dalian, China) according to the double enzyme digestion ligation method. The resulting proPsPIP2-4::GUS recombinant plasmid was transformed into *Agrobacterium tumefaciens* GV3101 competent cells (Weidi, Shanghai, China) by the freeze–thaw method, and transgenic tobacco was created via the *Agrobacterium*-mediated genetic transformation protocol as previously expounded [61,62]. The vector of pCAMBIA1300-pBI101 was also transformed into tobacco as the positive control plant. *Nicotiana benthamiana*, as the negative control (WT) plant, was utilized as the background for the tobacco transformation. Transformed tobacco seeds were screened on the Murashige and Skoog (MS) medium using 50 mg/L hygromycin (Hyg), and transformed tobacco seedlings were validated by sequencing of the genomic PCR products with the forward primer (5′-CACAAGAGACAAAGCGGTG-3′) in the *PsPIP2-4* promoter and the reverse primer (5′-TCGCGATCCAGACTGAATGC-3′) in the *GUS* reporter gene until the T3 homozygous lines, which were employed in subsequent studies. The primers used in PCR were listed in Table S1.

### 4.6. Transgenic Tobacco Stress Treatment

For stress treatments, the uniformly germinated seeds of WT, pCAMBIA1300-pBI101, and three proPsPIP2-4::GUS transgenic tobacco lines were initially sown on the MS medium in a controlled artificial climate chamber for 15 days. Then, the 15-day-old tobacco seedlings were transferred to the EP tubes containing 2 mL solutions with 6% PEG6000, 100 mM NaCl, and 100 µM MeJA solutions for 3 h or 12 h, respectively. In the parallel experiment, non-stressed WT, CAMBIA1300-pBI101, and proPsPIP2-4::GUS transgenic tobacco seedlings, which were transferred to the EP tubes containing 2 mL of water, were used as controls (CK). Transgenic tobacco seeds and seedlings were cultivated under 16 h light/8 h dark cycles at 25 °C and relative humidity of 60%. Each treatment contained three biological replicates. The whole seedlings of CK and stress-treated tobacco were utilized for subsequent GUS assays.

### 4.7. Measurement of GUS Activity

GUS staining and activity detection for the whole seedlings of WT, pCAMBIA1300-pBI101, and three proPsPIP2-4::GUS transgenic tobacco lines under different abiotic stresses were performed following the procedures as described previously [63,64,65,66]. For the GUS staining assay, the whole tobacco seedlings were immersed in the GUS dye solution (prepared just before use and stored in the dark) with 80 mM sodium phosphate (pH 7.0), 0.5 mM potassium ferricyanide, 0.5 mM potassium ferrocyanide, 10 mM EDTA, 1 mg/mL 5-bromo-4chloro-indolyl-b-D-glucuronide (X-Gluc), and 0.1% Triton X-100 at 37 °C for overnight cultivation. The GUS-stained seedlings were decolorized with 70% ethanol until the remaining green color on the leaves completely faded. The representative GUS-stained seedlings were observed and imaged using the Keyence VHX-500F digital microscope (Keyence, Osaka, Japan). For the GUS activity detection assay, the whole tobacco seedlings were quickly frozen in liquid nitrogen and then homogenized in the GUS extraction buffer with 50 mM sodium phosphate (pH 7.0), 10 mM EDTA (pH 8.0), 0.1% sodium lauryl-sarcosine, 0.1% Triton X-100, and 10 mM β-mercaptoethanol. The GUS reaction was conducted by the addition of 4-methylumbelliferyl-β-d-galactopyranoside (4-MUG) as a substrate and terminated by the addition of 0.2 M Na_2_CO_3_. Fluorescence was quantified with 4-methylumbelliferone (4-MU) as a standard using a fluorescence spectrophotometer (HITACHI F-4600, Tokyo, Japan) at excitation and emission wavelengths of 365 and 455 nm, respectively. Protein extract concentration was quantified with bovine serum albumin (BSA) as a standard. The means of nine replicates for GUS activities of picomole of 4-MU per minute per mg protein were calculated by Student’s *t*-test at a significant level of *p* < 0.01.

## 5. Conclusions

This study contributed to uncovering the biological function of the vegetable pea *PsPIP2-4* promoter in mediating abiotic stress responses. Protein structure and evolutionary analyses revealed that PsPIP2-4 shared the highest similarity to the PIP2-type *AQPs*. Expression pattern analyses suggested that *PsPIP2-4* possessed relatively high expression in leaves and remarkable expression changes in response to osmotic, salt, and MeJA stresses. Further, the *PsPIP2-4* promoter was isolated, and its activity was verified using the *GUS* reporter gene in transgenic tobacco plants. GUS staining and activity assays confirmed that the *PsPIP2-4* promoter was obviously expressed in the leaves of transgenic seedlings, especially in the guard cells, and strongly induced by osmotic, salt, and MeJA stresses. These findings clarified the regulatory function of the *PsPIP2-4* promoter in response to abiotic stresses and the application prospect of the *PsPIP2-4* promoter as a genetic tool for targeted expression of desired genes to improve the abiotic stress resistance of transgenic plants in the future.

## Figures and Tables

**Table 1:** Distribution of *cis*-acting elements in the *PsPIP2-4* promoter.

Element Name	Core Sequence	Number	Location (bp)		Function
			(+) Strand	(−) Strand	
CGTCA motif	CGTCA	1	191		MeJA responsiveness
TGACG motif	TGACG	1		191	MeJA responsiveness
DRE core	GCCGAC	1	233		Drought responsiveness
TC-rich repeats	ATTCTCTAAC	1	513		Defense and stress responsiveness

## Data Availability

The data involved in this research are listed in this article and its Supplementary Materials.

## Supplementary Materials

The following supporting information can be downloaded at: https://www.mdpi.com/article/10.3390/ijms252413574/s1.

## Abbreviations

ABA, abscisic acid; ACC, 1-aminocyclopropane-1-carboxylate; AQP, aquaporin; ar/R, aromatic/arginine; FPs, Froger’s positions; GA3, gibberellin A3; GUS, beta-glucuronidase; Hyg, hygromycin; MeJA, methyl jasmonate; 4-MU, 4-methylumbelliferone; 4-MUG, 4-methylumbelliferyl-β-d-galactopyranoside; MS, Murashige and Skoog; NIPs, NOD26-like intrinsic proteins; NJ, neighbor-joining; NPA, Asn-Pro-Ala; PEG, polyethylene glycol; PIP, plasma membrane intrinsic protein; qRT-PCR, quantitative real-time PCR; SIPs, small basic intrinsic proteins; TIPs, tonoplast intrinsic proteins; TM, trans-membrane; VIGS, virus-induced gene silencing; X-Gluc, 5-bromo-4chloro-indolyl-b-D-glucuronide; XIPs, the unrecognized X intrinsic proteins.
