# Supplementary material for: Promoter of Vegetable Pea PsPIP2-4 Responds to Abiotic Stresses in Transgenic Tobacco
Source: Int J Mol Sci. 2024 Dec 18;25(24):13574. doi: 10.3390/ijms252413574 (PMC11676869; doi:10.3390/ijms252413574)
Supplement: Supplementary file 1 [file ijms-25-13574-s001.zip › Supplementary Dataset S1.pdf]

Dataset S1. Fasta file of the promoter sequence for *PsPIP2-4* in vegetable pea.

1 CCATGAGGGT CAACACCGTG AAATTTGGCC ACATCAAGTT TCAGAATAAG ACGTTGAGGG

61 TCTTCCGTGT CGATGTTGTT GCGCGGGGTG ACAATTTTCG GTGATTGGGA ATGAAGCTTT

121 AAGGACTCAA CAGGGTGGTT GACTTTGTCC ATACTAAGTT GAAATGCTTC TGGAAACACG

181 AAAGGACTTG CGTCAAATCA TCCAATTTTC CGTTGGACGA ATGAGTGATA ACGCCGACCA

241 TTTTGGATTT GAAGGGAGAT GGAGGTCAGT GAGGAAAGCA CCACTGACAA AACTCCCTGT

301 GTTGCTCTGA TATTAGGCTT AGAGAAAACC CCTAAGAAAA ATACAAAAAG GGAACGAATT

361 TGACTTAATT TGATTCAATT CACGATGCTT CCAGCTTAAT AAATATAATG CAATTTCATA

421 ACAAAGAGAT TTGCAAAATT TAATGCAAAA TAGAATCTAA CAAACTTAAA TGAGTCATGC

481 TCTTCTAGAA CCGCTACCAA GTTAATCACA TAATTCTCAA ACTTTTTAAG AGACCTAATT

541 GTTCTCTTTC TCTTAAACCC TTGATTGTCA TTGTTTTTCT CTTCTATCAT TAGTCTCTTA

601 TCCTTACCAT TGAACCTTTA TATATCATAA TCTATTTAAT TGGTACAAAT ATCTCTATTT

661 ATTTTAATTC ATATATTATT TGAGATTAAA AGTAATGCAC TTTCAGTATA AAAAATATTT

721 CTTTTCATAT TATTTTTCTC CTACAATAAT ATATTTTCAT TACATTTACG GTAAATAAAA

781 AATGTGAAAA TAAGACTAGT TGAGAAAAAG AGTGTGAAAA TAAAAACACA TGAATTATGT

841 AGAATGTACT AGTATTATTT AGATCAATTA ATTAAAAAAT TTAAAAACTA TATTAACAAT

901 TAAAAATGGT AAATTTAAAC ATAGCTATTT TTGTTCGCAA AGGTGACAAT TCAGGAATAT

961 TCATTCAGTC TAGAAGTATG TGGCAAAATA AACAAAGAAT ACTTGCGAAT CTTGTGTAGA

1021 TGCATGACAC GATGGACATG AGAAGGATTA ACATGTGAGT GGGACCAACA CAAGAGACAA

1081 AGCGGTGGCT TGAGATATAC TAGTATTCCA GAGCAAAACA AAGGCGTAAC CGTTGGATCT

1141 GCGTTAAAGC ACTCCAAAAA GAGACAAAAC GCATCAATTT TCTAGTTATT ACGAAACATT

1201 TATCTTACTG TAGTACAAAA AACAAAAATA ATGATGACAA TAACATTGCT GAAATAACCT

1261 CTAAAAATAA CCATAACCCC CTCTTTCATG TTTTATGATT GTTGTAATCT TTTTAGCAGT

1321 ATCATATCTG TGCATAATTC TCTGCACATT TGATTTATCT CTGTAAAGTT ATTAACTGAG

1381 TTGGTTTAAC TGAAAATAAA TGTGTTGTAT GGTGGTATAA AAAACTACAT TTACTTCATT

1441 CCCTCAACAT TATTCTAATA ACAAAGAACA CTCACAAACG CCACACTCTC TCTAACTTC
