# Supplementary material for: Promoter of Vegetable Pea PsPIP2-4 Responds to Abiotic Stresses in Transgenic Tobacco
Source: Int J Mol Sci. 2024 Dec 18;25(24):13574. doi: 10.3390/ijms252413574 (PMC11676869; doi:10.3390/ijms252413574)
Supplement: Supplementary file 1 [file ijms-25-13574-s001.zip › Supplementary Figures S1-S3.pdf]

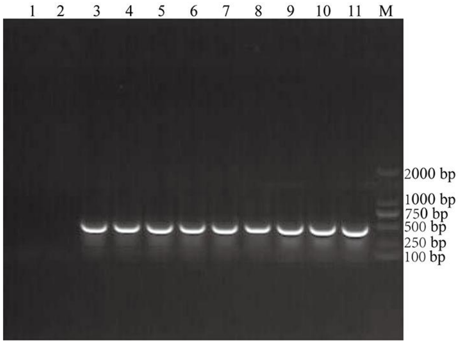

Figure S1. Agarose gel electrophoresis for genomic PCR products of tobacco seedlings. 1: the negative control (WT) tobacco seedling; 2: the positive control (pCAMBIA1300-pBI101) tobacco seedling ; 3-11: the positive T3 transgenic tobacco seedlings; M: DL2000 DNA Marker.

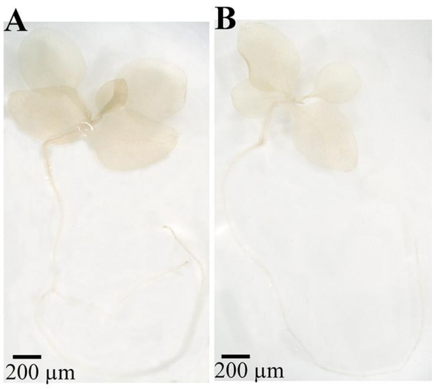

Figure S2. GUS histochemical staining for the negative control (WT) and positive control (pCAMBIA1300-pBI101) tobacco seedlings. (**A**) Negative control (WT) seedlings. (**B**) Positive control (pCAMBIA1300-pBI101) seedlings.

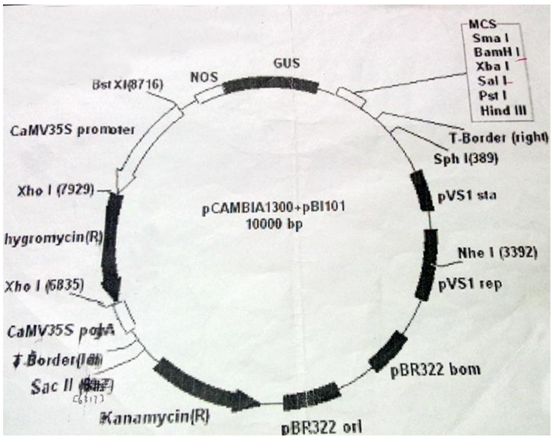

Figure S3. Schematic diagram of pCAMBIA1300-pBI101 vector with *Pst* I/*BamH* I restriction enzyme cleavage sites and *GUS* reporter gene.
