# Supplementary material for: Promoter of Vegetable Pea PsPIP2-4 Responds to Abiotic Stresses in Transgenic Tobacco
Source: Int J Mol Sci. 2024 Dec 18;25(24):13574. doi: 10.3390/ijms252413574 (PMC11676869; doi:10.3390/ijms252413574)
Supplement: Supplementary file 1 [file ijms-25-13574-s001.zip › Supplementary Table S1.pdf]

Table S1. List of primers used in this study.

	Gene-specific primers used for qRT-PCR of *PsPIP2-4*	
	Forward Primer	Reverse Primer
PsPIP2-4	AATCACAAACCGATCCAGCT	CCTAAACATTGAGCCACCATG
Psβtubulin	GCTCCCAGCAGTACAGGACTCT	TGGCATCCCACATTTGTTGA
PsEF1a	GATGCACCTGGACATCGTGAC	CTTAGGGGTGGTAGCATCCATCT
	Gene-specific primers used for cloning the promoter of *PsPIP2-4*	
	Forward Primer	Reverse Primer
proPsPIP2-4	CCATGAGGGTCAACACCGTG	GAAGTTAGAGAGAGTGTGG
	Gene-specific primers used for PCR of proPsPIP2-4::GUS transgenic tobacco	
	Forward Primer	Reverse Primer
proPsPIP2-4::GUS	CACAAGAGACAAAGCGGTG	TCGCGATCCAGACTGAATGC
